# Fusion proteins for treatment of retinal diseases: aflibercept, ziv-aflibercept, and conbercept

**DOI:** 10.1186/s40942-016-0026-y

**Published:** 2016-02-01

**Authors:** João Rafael de Oliveira Dias, Gabriel Costa de Andrade, Eduardo Amorim Novais, Michel Eid Farah, Eduardo Büchele Rodrigues

**Affiliations:** Department of Ophthalmology, Federal University of São Paulo-Paulista Medical School, Rua Botucatu, 821, 1st Floor, São Paulo, SP 04023-062 Brazil

**Keywords:** Fusion proteins, Aflibercept, Ziv-aflibercept, Conbercept, Vascular endothelial growth factor (VEGF), VEGF Trap Eye

## Abstract

In the last few years, monoclonal antibodies have revolutionized the treatment of retinal neovascular diseases. More recently, a different class of drugs, fusion proteins, has provided an alternative treatment strategy with pharmacological differences. In addition to commercially available aflibercept, two other drugs, ziv-aflibercept and conbercept, have been studied in antiangiogenic treatment of ocular diseases. In this scenario, a critical review of the currently available data regarding fusion proteins in ophthalmic diseases may be a timely and important contribution. Aflibercept, previously known as VEGF Trap Eye, is a fusion protein of VEGF receptors 1 and 2 and a treatment for several retinal diseases related to angiogenesis. It has firmly joined ranibizumab and bevacizumab as an important therapeutic option in the management of neovascular AMD-, DME- and RVO-associated macular edema. Ziv-aflibercept, a systemic chemotherapeutic agent approved for the treatment of metastatic colorectal cancer, has recently drawn attention because of its potential for intravitreal administration, since it was not associated with ERG-related signs of toxicity in an experimental study and in human case reports. Conbercept is a soluble receptor decoy that blocks all isoforms of VEGF-A, VEGF-B, VEGF-C, and PlGF, which has a high binding affinity for VEGF and a long half-life in vitreous. It has been studied in a phase three clinical trial and has shown efficacy and safety. This review discusses three fusion proteins that have been studied in ophthalmology, aflibercept, ziv-aflibercept and conbercept, with emphasis on their clinical application for the treatment of retinal diseases.

## Introduction

Vascular endothelial growth factor (VEGF) is a 36- to 46-kDa homodimeric glycoprotein that acts as an angiogenic cytokine, inducing mitosis [1]. It has six members, VEGF-A to -E, and placental growth factor (PlGF), of which VEGF-A is the most important cytokine involved in angiogenesis. There are several isoforms of VEGF-A in humans: VEGF_121_, VEGF_145_, VEGF_165_, VEGF_183_, VEGF_189_ and VEGF_206_. Of these, VEGF_165_ is the most common VEGF-A isoform and the most important for angiogenesis [2]. Three VEGF receptor (VEGFR) subtypes have been identified: VEGFR 1–3, among which VEGFR-1 binds VEGF with the highest affinity, while VEGFR-2 is the most important in angiogenesis [2].

Many cell types in the retina produce VEGF. These include the retinal pigment epithelium (RPE), vascular endothelial cells, pericytes, retinal neurons, Müller cells and astrocytes [3, 4]. VEGF is secreted by the RPE and retinal cells in response to hypoxia secondary to ischemic retinal disorders. Upregulation of VEGF results in angiogenesis, increased vascular permeability, and the production of pro-inflammatory cytokines [5].

Over the past decade, the use of intravitreal pharmacotherapy to block VEGF has become common and has significantly improved visual outcomes in patients with neovascular age-related macular degeneration (AMD), diabetic macular edema (DME) and retinal vein occlusion (RVO)-associated macular edema (ME) [6–8]. These retinal diseases are characterized by the production of increased levels of intraocular VEGF and development of ME resulting in dysfunction of central and sharp vision. VEGF also mediates the development of neovascularization in these conditions and may lead to severe irreversible vision loss. The administration of an anti-VEGF agent in the vitreous cavity of patients with these disorders lowers intraocular VEGF, reduces vascular permeability and is associated with arrested growth of and leakage from neovessels in choroidal neovascularization (CNV) [6, 9].

VEGF blockers used to treat eye diseases have included an aptamer, a humanized monoclonal antibody, an antibody fragment, and, more recently, cytokine traps [6, 10–12]. The purpose of this review is to provide an overview of three fusion proteins that have been studied for the treatment of retinal diseases: aflibercept, ziv-aflibercept and conbercept.

## Fusion proteins: history, chemistry, production, and biology

In 2002, Holash et al. published the first paper reporting the development and in vivo study of VEGF Trap for cancer treatment. VEGF Trap was created by fusing the first three immunoglobulin (Ig) domains of VEGFR-1 to the Fc region of human IgG1. Three additional VEGF traps were then engineered on the basis of that initial molecule: the VEGF Trap B1 (in which a highly basic 10-amino acid sequence was removed from the third Ig domain of the parental trap), the VEGF Trap B2 (in which the entire first Ig domain from VEGF Trap B1 was removed), and VEGF Trap R1R2 (the result of the fusion of the second Ig domain of VEGFR-1 with the third domain of VEGFR-2). These modifications enhanced R1R2 trap affinity for VEGF-A. The initial parental VEGF Trap had very high affinity for VEGF-A and PlGF, but was a strongly positively charged molecule that bound to the extracellular matrix in addition to VEGF-A and PlGF. Modifications resulted in a less positively charged molecule that retained high affinity for VEGF-A and VEGF-B as well as PlGF, but did not specifically bind to the extracellular matrix [13].

The current aflibercept, previously called VEGF Trap Eye, evolved from the parental VEGF Trap studied by Holash et al. This fully human protein consists of an all human amino acid sequence, which minimizes the potential for immunogenicity in human patients [13]. Aflibercept (Eylea; Regeneron, Tarrytown, NY, USA, and Bayer, Leverkusen, Germany) is a dimeric glycoprotein with a protein molecular weight of 96.9 kDa. It contains approximately 15 % glycosylation to give a total molecular weight of 115 kDa. As a designed molecule featuring optimal pharmacologic characteristics to inhibit intraocular VEGF, intravitreal aflibercept injection (IAI) offers improved binding affinity and superior pharmacokinetics in an iso-osmotic formulation. Aflibercept may have approximately 100-fold greater binding affinity for VEGF-A than does either bevacizumab or ranibizumab [9, 10]. It binds to all VEGF-A isoforms and the related VEGFR-1 ligands, VEGF-B and PlGF, and it is the only United States Food and Drug Administration (FDA)-approved VEGF Trap for intravitreal use [9, 14].

Ziv-aflibercept (Zaltrap; co-developed by Sanofi-Aventis and Regeneron Pharmaceuticals, Inc, Tarrytown, NY, USA), is identical to aflibercept, except for its excipients and higher osmolarity. While aflibercept is available in a single-use glass vial designed to provide 0.05 ml of 40 mg/ml solution (2 mg) for intravitreal injection, ziv-aflibercept is available as 100 mg per 4 ml (25 mg per ml) solution or 200 mg per 8 ml (25 mg per ml) solution, in a single-use vial. Aflibercept is iso-osmolar, whereas ziv-aflibercept is hyperosmolar (1000 mOsm/l) relative to the vitreous [15, 16].

In 2012, ziv-aflibercept received United States FDA approval for use in combination with FOLFIRI (folinic acid, fluorouracil and irinotecan) in patients with metastatic colorectal cancer that is resistant to or has progressed after oxaliplatin-based regimens such as FOLFOX (folinic acid, fluorouracil, oxaliplatin) [16]. Its intravitreal off-label use in humans was not associated with toxicity, inflammation or higher rate of cataract induction [11, 17, 18]. Although this drug has a higher osmolarity when compared to aflibercept, serum and intraocular osmolarity may not be significantly altered after intravitreal injection of ziv-aflibercept [19].

In 2008, Zhang et al. published the first study of conbercept (KH902) (Lumitin; Chengdu Kanghong Biotech, Ltd., Sichuan, People’s Republic of China) in an experimental CNV monkey model [20]. Conbercept is a full human DNA sequence with a molecular weight of 143 kDa produced with Chinese hamster ovary cells, and it combines ligand-binding elements taken from extracellular domain 2 of VEGF receptors 1 (Flt-1) and extracellular domain 3 and 4 of VEGF receptors 2 (KDR) fused to the Fc portion of human IgG1 [20–22]. This drug binds VEGF dimers in a 1:1 ratio with a “two-fisted grasp” that resembles the action of aflibercept. The difference between conbercept and aflibercept is that the former also contains domain 4 of VEGFR-2, which was proved in previous studies to be essential to the receptor [10, 23, 24]. Domain 4 does not participate in ligand binding but structure analysis of ligand-bound VEGFR has revealed that this domain might be involved in specific homotypic interactions of the ligand-bound receptor, stabilizing receptor dimers and locking VEGF to the receptor in a rigid manner [20]. A preclinical study suggested that conbercept may have an affinity 50-fold higher for VEGF compared to bevacizumab and that it could be equally more efficient in inhibiting the proliferation of human umbilical vein endothelial cells induced by VEGF [20]. Like aflibercept and ziv-aflibercept, conbercept shows strong antiangiogenetic effects by binding with high affinity and neutralizing VEGF-A, all its isoforms, and PlGF [12].

Comparative table shows the structural differences between the three fusion proteins available for treatment of retinal diseases (Table 1).

**Table 1:** Comparative table showing the structural differences between the three fusion proteins available for treatment of retinal diseases

	Aflibercept	Ziv-aflibercept	Conbercept
Structure	Fusion of the second domain of VEGFRs 1 and the third domain of VEGFR 2 to the Fc portion of human IgG1	Fusion of the second domain of VEGFRs 1 and the third domain of VEGFR 2 to the Fc portion of human IgG1	Fusion of the second domain of VEGFR1 and the third and fourth domains of VEGFR2 to the Fc portion of human IgG1
Mechanism of action	Binds to all isoforms of VEGF-A, VEGF-B and PIGF	Binds to all isoforms of VEGF-A, VEGF-B and PIGF	Binds with all isoforms of VEGF-A, VEGF-B, VEGF-C and PIGF
Concentration	40 mg/ml	25 mg/ml	10 mg/ml
Binding affinity to VEGF-A (165)	Kd 0,49 Pm	Kd 0,49 Pm	Kd 0,5 Pm
Molecular weight	115 kDa	115 kDa	143 kDa
Osmolarity	286 mOsm	1000 mOsm	Not published
Half-life	7, 1 days	7, 1 days	4, 2 days (rabbits)

## Clinical application of fusion proteins in ophthalmology for treatment of retinal diseases

### Aflibercept

The United States FDA approved Aflibercept in November 2011 for the treatment of neovascular AMD, in October 2014 for ME following RVO, and in March 2015 for the treatment of DME [9, 14, 25–27].

To date, large phase 3 studies have been conducted to evaluate the efficacy and safety of intravitreal aflibercept for exudative AMD. The VEGF Trap-Eye: Investigation of efficacy and safety in wet AMD (VIEW 1 and 2) recruited treatment-naive patients with neovascular AMD from 362 centers worldwide [14]. From baseline to week 52, patients received 0.5 mg intravitreal ranibizumab every 4 weeks (Rq4), 2 mg aflibercept every 4 weeks (2q4), 0.5 mg aflibercept every 4 weeks (0.5q4), or 2 mg aflibercept every 8 weeks (2q8) after 3 monthly injections. During weeks 52 through 96, patients received their original dosing assignment using an as-needed regimen with defined retreatment criteria and mandatory dosing at least every 12 weeks. All aflibercept and ranibizumab groups were equally effective in improving best-corrected visual acuity (BCVA) and maintaining BCVA (lost <15 letters from baseline) at 96 weeks. The 2q8 aflibercept group was similar to ranibizumab in visual acuity (VA) outcomes during 96 weeks, but with an average of 5 fewer injections [14]. Over the 2 years of treatment, a generally favorable safety profile was observed for both intravitreal aflibercept and ranibizumab. The incidence of ocular treatment-emergent adverse events (AE) was balanced across all treatment groups, with the most frequent events associated with the injection procedure, the underlying disease, the aging process, or a combination thereof. The incidences of arterial thromboembolic events and death were similar across all treatment groups [14].

In two parallel phase 3 DME studies, VISTA^DME^ and VIVID^DME^, eyes were randomized in a 1:1:1 ratio to receive either 2 mg IAI every 4 weeks (2q4), 2 mg IAI every 8 weeks after 5 initial monthly doses (from baseline to week 16) with sham injections on non-treatment visits (2q8), or macular laser photocoagulation at baseline and sham injections at every visit [7]. The results demonstrated that aflibercept given either every 4 or 8 weeks was superior to laser alone, and results in both showed significant VA gains and prevention of severe VA loss. The mean change from baseline BCVA in the 2q4 and 2q8 groups compared with the laser group was +12.5 ± 9.5 and +10.7 ± 8.2 letters vs +0.2 ± 12.5 letters in VISTA (P < 0.0001), and +10.5 ± 9.5 and +10.7 ± 9.3 letters vs +1.2 ± 10.6 letters (P < 0.0001) in VIVID, respectively [7]. The percentage of eyes in the laser group that lost ≥15 letters of vision replicated the 10 % loss reported by the early treatment diabetic retinopathy study (ETDRS) [7, 28]. Overall incidences of ocular and nonocular AE were similar across treatment groups [7].

The Diabetic Retinopathy Clinical Research Network (DRCR.net), sponsored by the National Institutes of Health, conducted a multicenter, randomized clinical trial at 89 clinical sites in the United States to compare the efficacy and safety of intravitreous aflibercept, bevacizumab, and ranibizumab for the treatment of DME causing decreased VA. The study drugs were injected into the study eyes at baseline and then every 4 weeks unless VA was 20/20 or better with a central subfield thickness below the eligibility threshold and there was no improvement or worsening in response to the past two injections. Laser photocoagulation therapy (focal, grid, or both) was initiated at or after the 24-week visit for persistent DME. Between August 2012, and August 2013, 660 participants were randomly assigned to receive aflibercept 2.0 mg (224 participants), bevacizumab 1.25 mg (218), or ranibizumab 0.3 mg (218). The median number of injections was 9 or 10 in the three groups. The mean improvement in the VA letter score at 1 year was greater with aflibercept than with bevacizumab or ranibizumab (13.3 vs 9.7 and 11.2, respectively; P < 0.001 for aflibercept vs bevacizumab and P = 0.03 for aflibercept vs ranibizumab), but the relative effect varied according to initial VA. At the 1-year visit, the central subfield thickness decreased, on average, by 169 ± 138 μm with aflibercept, 101 ± 121 μm with bevacizumab, and 147 ± 134 μm with ranibizumab. Injection-related infectious endophthalmitis occurred in one aflibercept-treated eye and one ranibizumab-treated eye (both nonstudy eyes) and no bevacizumab-treated eyes. Through 1 year, the rate of serious adverse events was similar in the three treatment groups (P = 0.40), as was the rate of hospitalization (P = 0.51) [29].

Intravitreal aflibercept has also been investigated for the treatment of ME secondary to central retinal vein occlusion (CRVO) in two parallel trials, the COPERNICUS and GALILEO studies, performed in the United States and in Europe and Asia/Pacific, respectively [8, 30, 31]. In the GALILEO study, patients were randomized to receive either 2 mg IAI or sham in the study eye once every 4 weeks for 20 weeks, for a total of six doses. From week 24 to week 48, patients in the IAI group were evaluated every 4 weeks and received aflibercept as needed or *pro re nata* (PRN) if they met prespecified retreatment criteria [8]. The proportion of patients who gained ≥ 15 letters in the intravitreal aflibercept and sham groups was 60.2 vs 22.1 % at week 24 (P < 0.0001), 60.2 vs 32.4 % at week 52 (P < 0.001), and 57.3 vs 29.4 % at week 76 (P < 0.001). The COPERNICUS study was a trial parallel to GALILEO, differing in the timing of the IAI. Patients received IAI 2 mg (IAI 2q4) or sham injections every 4 weeks up to week 24. During weeks 24 to 52, patients from both arms were evaluated monthly and received IAI PRN (IAI 2q4 + PRN and sham + IAI PRN). During weeks 52 to 100, patients were evaluated at least quarterly and received IAI PRN. The most frequent ocular severe adverse event (SAE) from baseline to week 100 was vitreous hemorrhage (0.9 vs 6.8 % in the IAI 2q4 + PRN and sham + IAI PRN groups, respectively) [8, 30, 31]. Overall, the COPERNICUS study demonstrated similar effects as those seen in GALILEO study in visual and anatomic improvements with IAIs after switching from monthly dosing [30].

The VIBRANT study was conducted to compare the efficacy and safety of IAI with macular grid laser photocoagulation for the treatment of ME after branch retinal vein occlusion (BRVO) [25]. It showed that monthly IAI provided significantly greater visual benefit and reduction in central retinal thickness (CRT) at 24 weeks than did grid laser. The proportion of eyes that gained ≥ 15 ETDRS letters from baseline at week 24 was 52.7 % in the IAI group compared with 26.7 % in the laser group (P = 0.0003). The mean improvement from baseline BCVA at week 24 was 17.0 ETDRS letters in the IAI group and 6.9 ETDRS letters in the laser group (P < 0.0001). The mean reduction in CRT from baseline at week 24 was 280.5 µm in the IAI group and 128.0 µm in the laser group (P < 0.0001). Traumatic cataract in an IAI patient was the only ocular SAE that occurred. The incidence of nonocular SAE was 8.8 % in the IAI group and 9.8 % in the laser group [25].

### Ziv-aflibercept

Ziv-aflibercept received FDA approval in August 2012 for the treatment of metastatic colorectal carcinoma that is resistant to or has progressed on an oxaliplatin-based regimen [16]. Due to its mechanism of action, there is growing interest in using intravitreal ziv-aflibercept as an antiangiogenic agent for ophthalmic VEGF-related diseases [17].

**Fig. 1:**
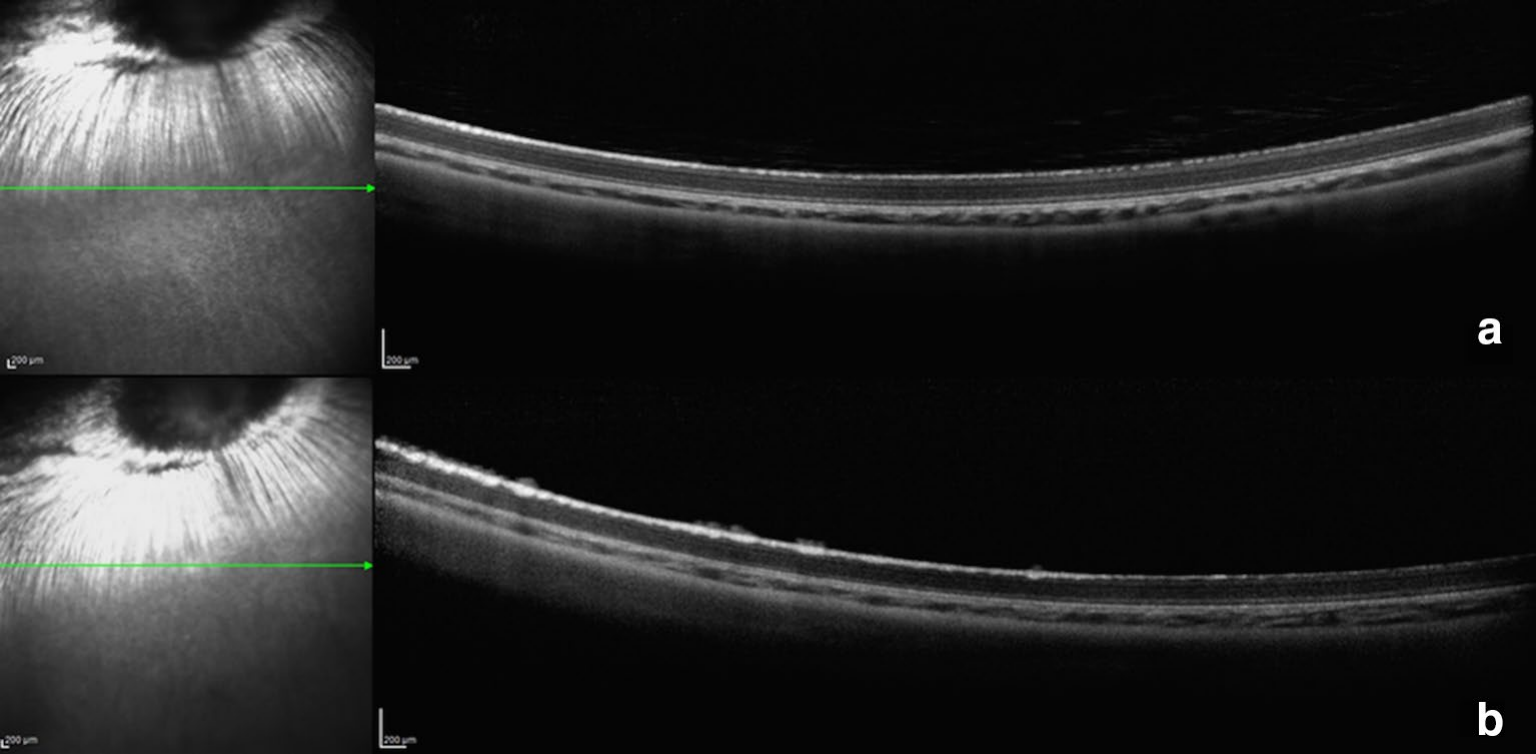
Spectral-domain OCT 7 days after intravitreal injection of 0.05 mL of ziv-aflibercept (25 mg/mL) (**a**) or aflibercept (40 mg/mL) (**b**) in two rabbits’ right eye

Malik et al. exposed human RPE cells for 24 h to four anti-VEGF drugs (bevacizumab, ranibizumab, aflibercept and ziv-aflibercept) at 1/2, 1, 2 and 10× clinical concentration. At clinical doses (1×) there was no decrease in cell viability in all four drug groups [32]. In an experimental study, nine rabbits were given an intravitreal injection of 0.05 ml ziv-aflibercept (25 mg/ml) [19]. There were no associated complications such as cataract and retinal detachment (RD). All eyes showed no signs of toxicity on funduscopy, optical coherence tomography (OCT) (Fig. 1), and full-field electroretinogram (ERG) 1 or 7 days after the procedure. There were also no changes in median baseline serum, vitreous, and aqueous osmolarities. Histology and transmission electron microscopy showed no major anatomic signs of toxicity, and no cytotoxic effect was observed in ARPE-19 cells exposed to clinical and 2× clinical concentration of ziv-aflibercept, which was also reported by Bababeygy et al. [19, 33]. del Carpio et al. showed that ziv-aflibercept was not detrimental to cell viability at a low dose (1/2×) and a clinical equivalent dose for MIO-M1 cells in vitro. However, twice the clini-cal dose of ziv-aflibercept reduced Müller cell viability, whereas this reduction at 2× clinical dose was not observed for ranibizumab, aflibercept, or bevacizumab [34].

**Fig. 2:**
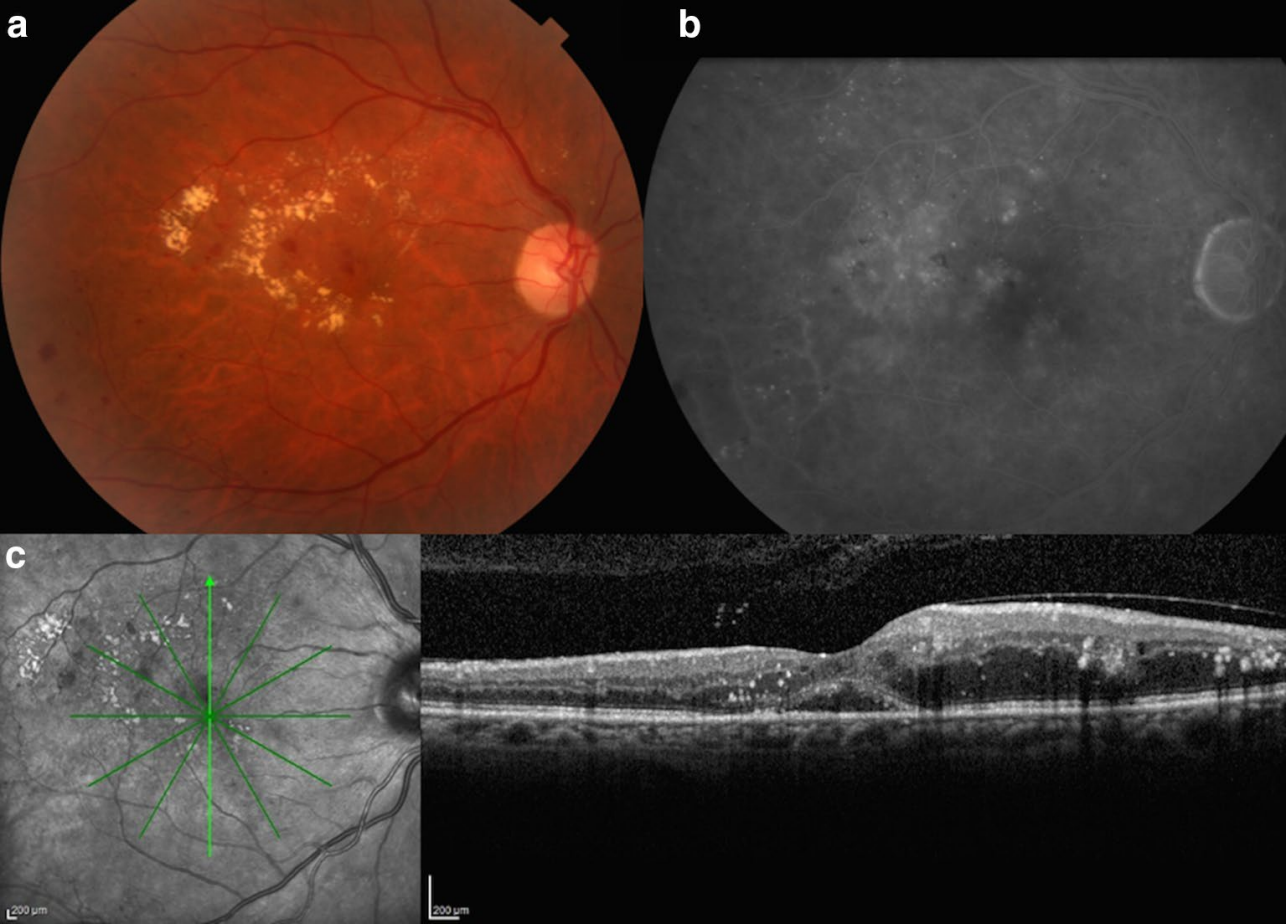
Baseline color fundus image (**a**), fluorescein angiography (**b**) and SD-OCT (**c**) of the right eye of a patient presenting DME. **a** At baseline, hard exudates and diffuse intraretinal fluid are seen in the perifoveal area. **b** At baseline, diffuse hyperfluorescence due to leakage (especially supero-temporally) and hypofluorescence due to non-perfusion (infero-temporally) are seen in the perifoveal area. **c** Cystoid spaces and subretinal and intraretinal fluid are seen in the foveal and perifoveal area

**Fig. 3:**
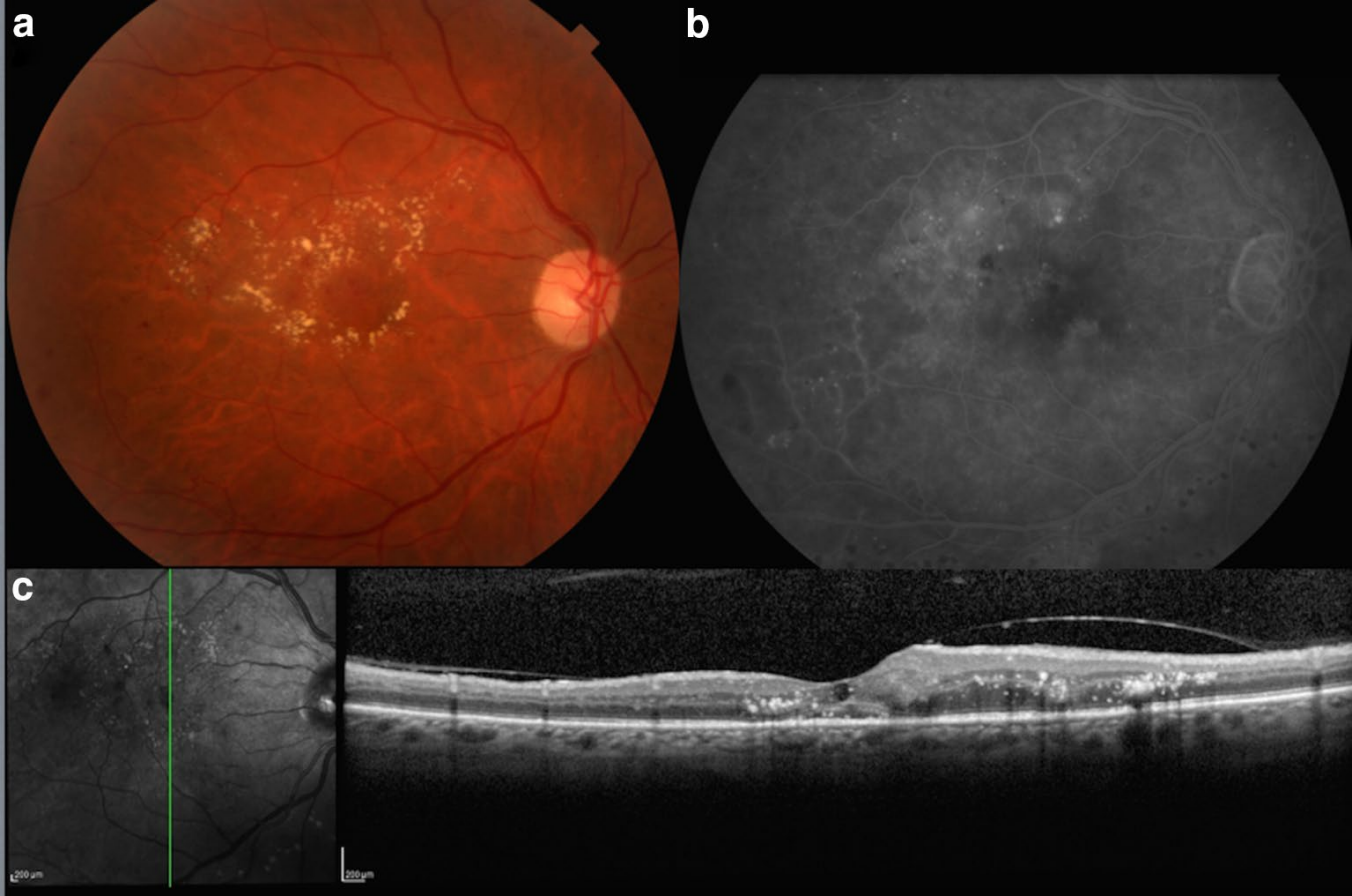
Color fundus image (**a)** fluorescein angiography (**b**) and SD-OCT (**c)** of the right eye of the same patient shown in Fig. 2. 4 weeks after the 3rd monthly injection of ziv-aflibercept. **a** 4 weeks after the third ziv-aflibercept injection, a decrease of intraretinal fluid and hard exudates is noticed in the perifoveal area of the right eye. **b** 4 weeks after the third ziv-aflibercept injection, a decrease of leakage is seen in the perifoveal area of the right eye. **c** 4 weeks after the third ziv-aflibercept injection, a decrease in the subretinal and intraretinal fluid is noticed

Recently, de Oliveira Dias et al. reported the first human intravitreal administration of ziv-aflibercept. A patient with refractory neovascular AMD was given 2 monthly intravitreal injections of 0.05 ml ziv-aflibercept (1.25 mg) and experienced subjective and objective improvement in VA with a decrease in intraretinal and subretinal fluid. No ERG changes were noticed when baseline and 30-day follow-up were compared. No AE were observed at any time point. The therapeutic dose of ziv-aflibercept was chosen on the basis of preliminary studies that showed efficacy of aflibercept at doses of 0.5–2 mg. The dose of 1.25 mg was chosen to keep the usual volume of 0.05 ml for intravitreal injections. Volumes larger than 0.05 ml may increase the risk of intraocular pressure [11]. Unpublished data from the DME study group from the Federal University of São Paulo showed regression of intraretinal fluid in a 49-year-old female patient presenting DME, 4 weeks after the third injection of 0.05 ml of ziv-aflibercept 25 mg/ml (1.25 mg) (Figs. 2 and 3 a, b, c). VA improved from 20/100 to 20/40, 4 weeks after the third injection of ziv-aflibercept.

Mansour et al. presented recent data on four patients with neovascular AMD and two patients with DME who received one intravitreal injection of 0.05 ml of ziv-aflibercept (1.25 mg). All six patients showed evidence of improvement in VA with no signs of intraocular inflammation, change in lens status or retinal toxicity [17]. Chhablani also reported the intravitreal injection of 1.25 mg intravitreal ziv-aflibercept in a patient presenting with bilateral ME secondary to CRVO, who had already received 12 and 13 anti-VEGF injections in the right and left eye, respectively, along with one intravitreal triamcinolone injection and peripheral panretinal photocoagulation in both eyes. At 1-month follow-up of intravitreal ziv-aflibercept injection in both eyes, the patient experienced an improvement in VA along with CRT decrease. He did not have any symptoms of blurred vision or ocular pain related to injection or any signs of inflammation/toxicity [35].

### Conbercept

Conbercept (KH902) has been studied in a phase 3 clinical trial and was approved to treat neovascular AMD by the China State FDA in December 2013. Conbercept has not yet reached the market in other countries [36].

After preclinical results showing the antiangiogenetic effects of conbercept, there has been a growing interest in using this drug intravitreally to treat VEGF-related ophthalmic diseases [12, 20, 36]. The AURORA study was a 12-month, randomized, double-masked, controlled-dose, and interval-ranging phase two clinical trial that took place at nine sites in China; it was designed as a superiority trial to assess the safety and efficacy of different dosing regimens. Eligible patients were randomized 1:1 to 0.5- or 2.0-mg treatment groups. Initially, all patients received monthly intravitreal injections of conbercept for a total of three injections. After three loading doses of monthly intravitreal conbercept injection, the patients were then randomized into the monthly (Q1 M) or PRN group. One hundred and twenty-two patients were enrolled. At 12 months, mean improvements in BCVA from baseline were 14.31, 9.31, 12.42, and 15.43 letters for the 0.5-mg PRN, 0.5-mg Q1 M, 2.0-mg PRN, and 2.0-mg Q1 M regimens, respectively. A reduction in CRT was also detected. At 12 months, mean reductions in CRT in the four regimens were 119.8, 129.7, 152.1, and 170.8 μm, respectively. At 12 months follow-up, no significant differences in BCVA or anatomic outcomes were found between the groups, regardless of the dose or dosing regimen. Overall, conbercept was well tolerated, and the incidence of ocular AE was low. The most common AE were usually caused by the intravitreal injection procedure and disappeared with or without treatment. RD did not occur in this study. A case of hepatitis suspected of being drug induced was identified and thought to be caused by an oral supplement, and the hepatitis was categorized as having no relationship with the study drug [12].

A phase 3 trial with intravitreal conbercept for exudative AMD was completed in 2013, and the results were announced at Angiogenesis, Exudation, and Degeneration in 2014 [37, 38]. In this trial, patients in the treatment group received three fixed monthly injections and then two sham injections monthly followed by conbercept injection every 3 months up to 12 months (intravitreal con-bercept at 0, 1, 2, 5, 8, and 11 months). Patients in the sham injection group were given three monthly sham injections and then crossed over to the treatment group. Both groups had a 12-month follow-up period. The report from the Angiogenesis, Exudation, and Degeneration meeting in 2014 showed that patients exhibited a mean change in BCVA of +10 letters at 12 months. Morphologic changes observed by OCT exhibited a significant reduction in central subfield thickness of 79 μm in the treatment groups at 3 months, whereas the sham injection group experienced a decrease of 44 μm, which was not significant. When the sham injection group crossed over to the treatment group, similar results were also observed at 12 months on OCT and fluorescein angiography. A reduction in subretinal fluid maximum height at 3 months was seen in the treatment group. After crossing over to the treatment group, 93 % of participants had less than 320 μm central subfield thickness at 12 months [20, 37, 38].

## Conclusions

Fusion proteins are a promising treatment for ocular diseases related to angiogenesis. They are fully human and may have higher affinity, binding VEGF more tightly than native receptors or monoclonal antibodies. They block all VEGF-A isoforms, VEGF-B, and PlGF, and should penetrate all retinal layers.

Aflibercept, the only United Stated FDA-approved fusion protein for ocular diseases, has firmly joined ranibizumab and bevacizumab as an important therapeutic option in the management of neovascular AMD, DME and RVO. Ziv-aflibercept, a systemic chemotherapeutic agent approved for the treatment of metastatic colorectal cancer, has been recently studied as a lower cost alternative for the treatment of retinal diseases. Although no signs of retinal toxicity were found in patients subjected to intravitreal ziv-aflibercept injection, its safety and efficacy still remain to be proved in larger studies. Conbercept has also been studied for the treatment of exudative AMD in a phase 3 study, showing no signs of retinal toxicity, along with an improvement in VA. More data are necessary to evaluate the retinal safety and efficacy of intravitreal ziv-aflibercept and conbercept.

## Abbreviations

VEGF: vascular endothelial growth factor
PlGF: placental growth factor
VEGFR: vascular endothelial growth factor receptor
RPE: retinal pigment epithelium
AMD: age-related macular degeneration
DME: diabetic macular edema
RVO: retinal vein occlusion
ME: macular edema
CNV: choroidal neovascularization
Ig: immunoglobulin
kDa: kilo Daltons
IAI: intravitreal aflibercept injection
FDA: Food and Drug Administration
BCVA: best-corrected visual acuity
VA: visual acuity
AE: adverse event
ETDRS: early treatment diabetic retinopathy study
CRVO: central retinal vein occlusion
PRN: pro re nata
CRT: central retinal thickness
SAE: serious adverse event
BRVO: branch retinal vein occlusion
RD: retinal detachment
OCT: optical coherence tomography
ERG: electroretinogram
